# Curcumin as a Therapeutic Agent in Dementia: A Mini Systematic Review of Human Studies

**DOI:** 10.1155/2014/174282

**Published:** 2014-01-22

**Authors:** Natascia Brondino, Simona Re, Annalisa Boldrini, Antonella Cuccomarino, Niccolò Lanati, Francesco Barale, Pierluigi Politi

**Affiliations:** ^1^Department of Brain and Behavioral Sciences, University of Pavia, Via Bassi 21, 27100 Pavia, Italy; ^2^Department of Public Health, University of Pavia, Viale Golgi 19, 27100 Pavia, Italy

## Abstract

Dementia is a leading health problem worldwide, with Alzheimer's disease (AD) representing up to 60% of all dementia cases. A growing interest has recently risen on the potential use of natural molecules in this condition. Curcumin is a polyphenolic compound traditionally used in Indian medicine. Several *in vitro* and *in vivo* studies have found a protective effect of curcumin in AD. In the present systematic review we aimed to evaluate the state-of-the-art of clinical trials of curcumin in AD. We retrieved three published studies, while there are several ongoing clinical trials. To date there is insufficient evidence to suggest the use of curcumin in dementia patients. Of note, short-term use of curcumin appears to be safe. Several reasons could be responsible for the discrepancy between *in vitro* and *in vivo* findings and human trials, such as low bioavailability and poor study design.

## 1. Introduction

The prevalence of dementia in 2010 reached 5%–7%, with 35.6 million people affected, and this figure may at least double in the next few decades [1]. Alzheimer's disease (AD) represents nearly 60% of all dementia patients [2]. AD is characterized by a progressive deterioration of cognitive function, loss of memory, and behavioral and personality changes. Of note, mild cognitive impairment (MCI) is a condition which is characterized by memory impairment beyond that expected for age and education [3]. Patients with MCI do not meet dementia diagnostic criteria, but are at high risk for progression to AD [4]. The principal histological features of AD are the senile plaques, the neurofibrillary tangles, and the presence of a conspicuous neuronal loss. Two proteins play a key role in the pathogenesis of AD: amyloid-*β*-protein (A*β*) and tau, which are the main constituent of senile plaques and neurofibrillary tangles, respectively [5, 6]. In particular, A*β* is derived from the cleavage of the amyloid precursor protein (APP) and aggregates as oligomers and fibrils in the brain parenchyma and in the cerebral vasculature, causing significant neuronal loss and synaptic impairment. Of note, A*β* oligomers appear to be more toxic than fibrillary aggregates and senile plaques. A*β*40 and A*β*42 are the two principal forms of A*β*, with A*β*42 being more subjected to aggregation [7]. Additionally, A*β* promotes hyperphosphorilation of tau: hyperphosphorylated tau aggregates to form neurofibrillary tangles and disrupts the mitochondrial membrane, leading to apoptotic cell death [8]. Despite the huge amount of data regarding the pathogenesis of AD, a limited number of therapeutic drugs have yet been developed. Recently, there is an increasing interest in natural antioxidants contained in food, which appear to have less side effects and higher tolerability.

Curcumin is derived from the rhizome of the *Curcuma longa*. It is contained in culinary curry and used as a coloring agent in food. Traditional Indian medicine considered this polyphenolic compound as an effective therapy for several pathological conditions, ranging from asthma to epilepsy, from gall stone to diabetic wound healing [9].

The hypothesis of a potential therapeutic role of curcumin in dementia originates from epidemiological data. In 2000, Ganguli and coworkers [10] reported a lower prevalence of AD in the Indian population, who consumes a diet rich in curcumin as a part of curry, compared to the USA population. Recently, Ng and colleagues [11] found that elderly healthy individuals who consume more frequently curry show a better cognitive performance. Moving from these preliminary observations, several *in vitro* and *in vivo* studies were conducted in order to find a protective effect of curcumin in AD. *In vitro* studies demonstrated a neuroprotective and antioxidant effect of curcumin, which appeared to be greater than that of tocopherol. In particular, curcumin protects neuron-like PC12 rat cells and umbilical endothelial cells against A*β* toxicity and reduces tau hyperphsphorylation [12], promotes A*β* uptake from macrophages of AD patients [13], and dose-dependently reduces fibril formation and extension, also destabilizing preformed A*β* fibrils [14–16]. Additionally, curcumin decreases levels of A*β*-induced radical oxygen species [17] and inhibits APP cleavage [18]. Of note, in rat hippocampal slices treated with A*β* oligomers, curcumin restores synaptic plasticity, by enhancing longterm potentiation [19]. The performance of *in vivo* studies has been hampered by the low bioavailability of curcumin. In fact, in rats, oral curcumin is poorly absorbed and undergoes extensive metabolization and glucuronidation in the intestinal wall and in the liver [20]. However, Lim and coworkers [21] orally administered a low dose of dietary curcumin (160 ppm) to an Alzheimer transgenic mouse model (Tg2576) for six months and observed reduced inflammation and oxidative stress in the brain. In particular the authors found a decrease of A*β* levels and of the number of plaques in different brain areas. Of note, a higher dose of dietary curcumin (5000 ppm) did not reduce A*β* levels. Frautschy and colleagues [22] infused A*β* to induce deposits and neurodegeneration in rats, in order to mimic Alzheimer histological alterations. Dietary curcumin (2000 ppm) succeeded in reducing oxidative damage and increased microglial reaction near A*β* deposits. Additionally, low doses of curcumin (160 ppm) avoided the occurrence of spatial memory impairment in rat treated with A*β* infusion. Subsequently, a study from Yang et al. [16] was performed in Tg2576 mice; they showed, in accordance with previous data, that curcumin reduced A*β* oligomer and fibril formation. In another study [23] conducted in the same mouse model, low doses of curcumin (500 ppm) orally administered for four months determined a reduction in plaque burden and A*β* levels. Using another Alzheimer mouse model (APP-swe/PS1dE9), Garcia-Alloza and coworkers [24] demonstrated an enhanced clearance of A*β* deposit in mouse brain after the intravenous administration of curcumin (7.7 mg/kg/day) for 7 days. Of note, they used multiphoton microscopy *in vivo* and found that curcumin crossed the blood brain barrier. Recently Hamaguchi et al. [25] observed that a dose of 5000 ppm of curcumin increased A*β* monomer levels while it decreased A*β* oligomer concentration in Tg2576 mice. However, they did not find any effect of curcumin on A*β* deposition in brain tissues. The authors hypothesized that curcumin may prevent A*β* polymerization but may not have any effect on A*β* deposition.

In humans, curcumin seems to have a good safety profile. Studies in cancer patients reported no toxicity in 25 patients taking oral curcumin (from 500 to 8000 mg/day) for three months [26]. From a pharmacokinetic point of view, after oral administration, serum levels of curcumin peaked after 1-2 h and declined within 12 h after intake. The range for serum concentration was between 0.51 ± 0.11 *μ*M at a dose of 4000 mg/day and 1.77 ± 1.87 *μ*M at a dose of 8000 mg/day [26]. Recently, a dose escalation study was conducted in healthy volunteers [27]. Curcumin oral dose ranged from 500 to 12000 mg, and serious adverse events were not reported. Only 30% of subjects experienced a minor toxicity (headache, diarrhea, and rash) which was not dose-related [27]. There is a dearth of evidence about the outcome of curcumin use in patients with cognitive decline. Of note, the majority of clinical studies on curcumin have focused on the effect of this natural compound on cancer. The aim of the present systematic review was to evaluate the state-of-the-art of the efficacy of curcumin in patients with dementia.

## 2. Materials and Methods

In July 2013, we searched the following databases: MEDLINE, EMBASE, and the Cochrane Database of Systematic Reviews. The search terms were: curcumin* (curcumin OR curcuminoids) and dementia (dementia or cognitive impairment or Alzheimer). All search terms were searched individually in each database and combined together. The search strategy had no time restriction but was limited to articles in English, Italian, French, Spanish, and German. Additionally, all recovered papers were reviewed for further relevant references.

We selected clinical trials, yielding primary results on the effects of the administration of curcumin in patients with dementia. Dementia (particularly Alzheimer's disease) was defined according to internationally valid diagnostic criteria such as the International Classification of Diseases (ICD) or the Diagnostic and Statistical Manual of Mental Disorders (DSM). We included randomized clinical trials as well as open-label trials.

Two researchers (Natascia Brondino and Annalisa Boldrini) independently reviewed all information about the articles provided by the databases. Any discrepancies were solved by consensus. We assessed the methodological quality of the included studies according to the criteria developed by the Cochrane Collaboration. We extracted data using a format which included study design, number of subjects, curcumin dose, additional medication, adverse events and main findings.

## 3. Results

**Figure 1:**
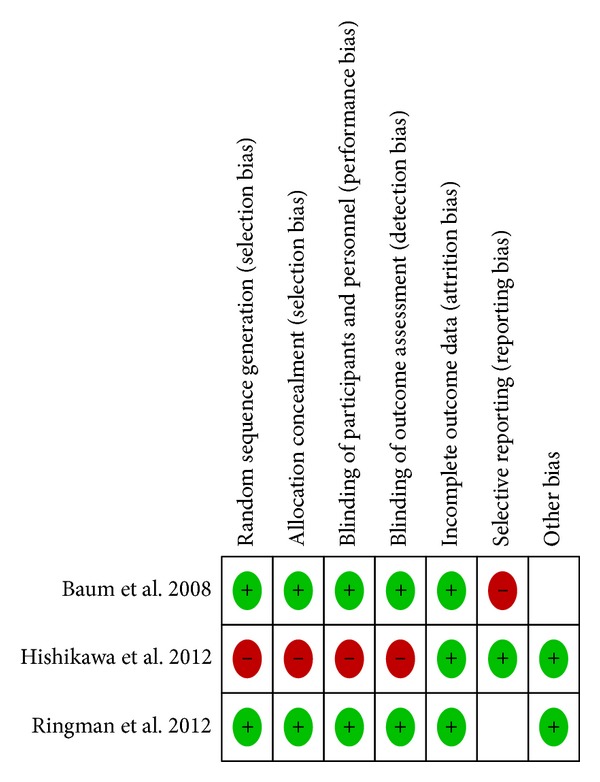
Methodological quality of the included studies.

Our search strategy yielded 984 citations. After screening of title and abstract, only 31 were retained for full-text examination. Only three studies fulfilled the inclusion criteria (Table 1). Overall quality of the included studies is depicted in Figure 1.

In 2008, Baum et al. [28] performed a randomized, double-blind, placebo-controlled study. They enrolled 34 patients with Alzheimer's disease. Each subject randomly received either curcumin at two different doses (1 g/day or 4 g/day) or placebo (4 g/day) for six months. Curcumin was either a capsule or a powder to be mixed with food. All subjects also received 120 mg/day of standardized gingko biloba leaf extract. Patients were allowed to continue any previous medications (except anticoagulant or antiplatelet drugs). The main outcome measure was the Mini-Mental State Examination (MMSE) score change between the baseline and the follow-up assessment. The authors did not observe any significant difference between curcumin and placebo. Additionally, curcumin treatment did not reduce serum A*β*40 levels. Of note, the curcumin group showed an increase in vitamin E levels. No serious side effect was reported. Another randomized, double-blind, placebo-controlled study was carried on in 2012. Ringman and colleagues [29] recruited 36 patients with dementia which randomly received 2 g/day or 4 g/day of Curcumin C3 Complex in two divided doses or placebo for 24 weeks. Curcumin C3 Complex is powder plant extract (Sabinsa Corporation, Piscataway, NJ, USA) and contains 95% of curcuminoids (consisting of 70% to 80% curcumin, 15% to 25% demethoxycurcumin, and 2.5% to 6.5% bisdemethoxycurcumin). After 24 weeks, the trial was extended to 48 weeks as an open-label trial in which patients who received placebo were randomly assigned to 2 g/day or 4 g/day of Curcumin C3 Complex, while patients on treatment continued with the same dose assigned at baseline. Primary outcomes were changes at the Alzheimer's Disease Assessment Scale, cognitive subportion (ADAS-Cog) at 24 weeks, and tolerability at 48 weeks. Secondary outcome measures were change at the Neuropsychiatric Inventory (NPI), the Alzheimer's Disease Cooperative Study Activities of Daily Living (ADCS-ADL), and the MMSE. Additionally, the authors evaluated modification in plasma and cerebrospinal fluid (CSF) markers. The authors did not observe any significant difference between treatment groups in change in ADAS-Cog, NPI, ADCS-ADL, or MMSE scores. Plasma and CFS levels of A*β*40–A*β*42 or tau were not different between treatment groups. No serious adverse event was reported. Of note, plasma levels of curcumin were undetectable after single doses; this is consistent with the low bioavailability of oral curcumin. The authors stated that, given the small sample size and the short study duration, they did not expect any significant effect of curcumin on clinical variables. Moreover, differences in disease severity at baseline may have biased the results; for instance, curcumin might have exerted a major impact in a subgroup (i.e., patients with mild conditions). In 2012, Hishikawa et al. [30] reported a case study of three dementia patients treated with 100 mg/day of curcumin. All three patients experienced a decreased in NPI-questionnaire brief version (NPI-Q) score (particularly, reduction in agitation, irritability, anxiety, and apathy) after 12 weeks of therapy. One patient with moderate cognitive decline (12/30 on MMSE) improved his MMSE score of five points. Of note, all patients were on anti-dementia medication (donepezil) before starting curcumin.

Interestingly, there are several ongoing clinical trials evaluating the efficacy of curcumin in AD or MCI. One study (NCT00595582) [31] has been completed but it did not produce significant results; the authors recruited 10 subjects with MCI which randomly received 5.4 g/day of curcumin + bioperidine or placebo for 24 months. Unfortunately, all participants did not terminate the study. Additionally, a phase II study (NCT01001637) [32] comparing curcumin (4 g/day or 6 g/day) and placebo is still recruiting patients with AD. All participants will take the active compound or the placebo for 60 days and the cognitive performance and A*β* plasma levels will be evaluated. A larger randomized, double-blind, placebo-controlled clinical trial (NCT01383161) [33] is being designed in order to test the effect of curcumin supplement (90 mg twice daily) in 132 subjects with memory complaints (MCI or age-associated memory impairment, not overt dementia). Participants will be treated up to 18 months and will be evaluated at three different times (6, 12, 18 months) after baseline assessment. Primary outcome measure will be the change in cognitive performance at the three time points. Secondary outcome measures will be imaging and plasma biomarker levels modification. The study appears to be well-designed and more restrictive on medication exclusion criteria (only aspirin is permitted). Another trial (NCT01811381) [34] will evaluate the effect of curcumin and yoga in 80 patients with MCI. For the first 6 months of the study, participants will assume 800 mg/day of either curcumin or placebo. From six to 12 months after baseline, the authors design a four arm study in which each of the two groups (curcumin or placebo) will be randomly split into two subgroups: one subgroup will attend aerobic yoga exercise program (2 classes of 1 hour duration and 2 home practices of 30-minute duration per week) while the other will attend a nonaerobic yoga program (with the same schedule as the aerobic yoga exercise program). Primary outcome will be the change on the NPI-Q score, while secondarily the authors will evaluate imaging changes in all participants. In Australia, a randomized double-blind placebo controlled trial [35] will soon start to recruit patients with dementia (sample size: 200) which will be treated with oral curcumin titrated up to 1500 mg/day. The primary outcome is the prevention of cognitive decline in the curcumin treated group.

## 4. Discussion

Several preclinical studies provided evidence supporting the efficacy of curcumin against AD pathophysiological features (i.e., A*β* polymerization and deposition). Unfortunately, to date, only few clinical trials have been completed, yielding negative or inconclusive results. Potential reasons for the discrepancy between *in vivo* and *in vitro* tests and human studies are numerous. Firstly, curcumin possesses poor oral bioavailability due to low absorption and rapid hepatic and intestinal metabolization [20, 36]. This lead to low or undetectable plasma levels after single oral dose and, subsequently, potentially insufficient brain levels [29]. To overcome this issue, new formulations are currently being developed. For instance, piperidine (found in black pepper) may act as UDP-glucuronosyltransferase inhibitor. If administered with curcumin, piperidine may block enteric and hepatic glucuronidation, thus resulting in higher curcumin plasma and tissue levels. Of note, in healthy subjects receiving a dose of 2 g curcumin alone, serum levels were undetectable. Piperine determined an increase in bioavailability of 2000% [37]. Other promising directions come from nanoparticles, micelles, and liposomes, which represent optimal delivery systems for hydrophobic substances as curcumin. In particular, after administration of curcumin dispersed with colloidal nanoparticles, bioavailability showed a dramatic increase compared to curcumin alone [38] or curcumin combined with piperidine [39]. Of note, curcumin nanoparticles were effective in Alzheimer Tg2576 transgenic mouse model [40]. Similar increases in bioavailability were observed if curcumin-phospholipid complex or polymeric micellar curcumin were administered [41, 42]. Another potential reason for the lack of positive results in clinical studies is that these trials were highly underpowered [28, 29]. Ongoing trials with larger sample size may show more reliable findings. Additionally, follow-up period could have been too short to detect potential changes in dementia symptoms and progression. In fact, AD symptoms usually become evident after a long time from the beginning of the disease and therefore brain tissues are usually more extensively affected than in animal model [43]. According to its pharmacodynamic properties, curcumin seems to act more as a neuroprotective agent than as a reversal medication. Thus, it is possible that curcumin treatment may represent a prevention and not a treatment.

In conclusion, to date there is insufficient evidence to suggest the use of curcumin in dementia patients. Of note, short-term use of curcumin appears to be safe; longer studies are needed to elucidate the potential presence of chronic toxicity in humans. Hopefully, future findings from ongoing clinical trials will shade more light on the potential therapeutic efficacy of curcumin in dementia.

## Figures and Tables

**Table 1:** Clinical studies of curcumin use in Alzheimer's disease.

Study ID	Study design	Sample size	Follow-up period	Curcumin dose	Other medication	Main findings	Adverse events	Current status
Completed studies								
Baum et al. 2008 [28]	Randomized, double-blind, placebo controlled	36	6 months	1 g/day or 4 g/day	Gingko biloba standardized leaf extract 120 mg/day, other medication not reported	No differences between curcumin and placebo	No differences between placebo and both curcumin dose groups	Completed and published
Ringman et al. 2012 [29]	Randomized, double-blind, placebo controlled	36	24 weeks + 48 weeks open-label	2 g/day or 4 g/day	Acetylcholinesterase inhibitors and memantine allowed	No differences between curcumin and placebo	No differences between placebo and curcumin	Completed and published
Hishikawa et al. 2012 [30]	Case study, open-label	3	1 year	100 mg/day	Donepezil (dose not reported)	Increase in the NPI-Q score	Not reported	Completed and published
Ongoing trials								
NCT00595582	Open-label	10	24 months	5.4 g/day	Bioperidine	All patients did not terminate the study	Dyspepsia (20% of the sample)	Completed
NCT01001637	Randomized, double-blind, placebo controlled	26	2 months	4 g/day or 6 g/day	Allowed stable doses of concomitant medications	—	—	Still recruiting
NCT01383161	Randomized, double-blind, placebo controlled	132	18 months	180 mg/day	Permitted only aspirin (81 mg/die)	—	—	Still recruiting
NCT01811381	Randomized, double-blind, placebo controlled	80	12 months	800 mg/day	Not allowed treatment for cognitive impairment (i.e. cholinesterase inhibitor, memantine) < 6 months prior to study enrollment	—	—	Recruiting will start in September 2013
ACTRN12613000681752	Randomized, double-blind, placebo controlled	200	12 months	500 mg/day for 2 weeks, then 1,000 mg/day for other 2 weeks and then 1500 mg/day onwards	Not allowed warfarin	—	—	Not yet recruiting

Legend. Neuropsychiatric Inventory Questionnaire: NPI-Q.
